# Identification of potential immune-related genes and infiltrations in temporomandibular joint osteoarthritis

**DOI:** 10.1097/MS9.0000000000002682

**Published:** 2024-10-23

**Authors:** Mengjiao Zhu, Min Xing, Ruinan Sun, Minhui Li, Wenhao Qian, Mingyue Fan

**Affiliations:** a Department of Orthodontics, Shanghai Xuhui District Dental Center, Shanghai, China; b Dental Laboratory, Shanghai Xuhui District Dental Center, Shanghai, China; c Department of Endodontics, Stomatological Hospital and Dental School, Tongji University, Shanghai Engineering Research Center of Tooth Restoration and Regeneration, Tongji Research Institute of Stomatology, Shanghai, China; d Department of vascular surgery, Institute of Vascular Surgery, Zhongshan Hospital, Fudan University, Shanghai, China; e Department of Oral Implantology, Shanghai Xuhui District Dental Center, Shanghai, China

**Keywords:** bioinformatics analysis, immune infiltration, inflammatory cytokines, temporomandibular joint osteoarthritis

## Abstract

**Objective:**

The aim of this study was to investigate the potential inflammatory cytokines and chemokines markers for temporomandibular joint osteoarthritis (TMJOA) diagnosis using a bioinformatics analysis.

**Methods:**

The differentially expressed genes of mRNA (DEGs) and transcripts of lncRNA (DETs) were identified between TMJOA samples and normal controls curated from GSE205389 by the “DESeq. 2” R package. KEGG and GO were conducted using the R package “ggplot2” and “clusterProfiler”. A PPI network was constructed to identify hub genes by using the STRING and Cytoscape. The co-expression network was constructed between mRNA and lncRNA to check the potential regulation and function of lncRNA on protein-coding genes. Finally, the immune cell infiltration analysis was conducted with CIBERSORTx and confirmed with xCells.

**Results:**

The authors identified 171 DEGs and DETs, of which the DEGs were closely related to immune response, T-cell activation, cytokine-cytokine-receptor interaction, and the muscle system process. PPI network of the DEGs screened the top 10 hub genes, including *IL6, IL1B, IL10, CCL2, CCL5, CXCL1, CXCL10, ICAM1, CSF1* and *MMP1*. Additionally, the immune cell infiltration analysis showed that CD8^+^ T cells, M1 macrophage and B cells infiltration were increased in TMJOA samples. Finally, the authors demonstrated that the co-expression between mRNA and lncRNA was mainly enriched in inflammatory and muscle-related pathways.

**Conclusions:**

The authors found that immune and muscle system-related pathways as well as the immune infiltration played a significant role in the TMJOA development. Additionally, inflammatory cytokines and chemokines could be crucial markers for early-stage TMJOA diagnosis and personalized treatment strategies.

## Introduction

Highlights

- Carry out a bioinformatics analysis of synovium samples from TMJOA patients and their controls.
- Contribute to the TMJOA disease diagnosis and mechanism exploration.
- Help to identify targets of immunotherapy for TMJOA patients.

Temporomandibular joint (TMJ) is a synovial joint that connects the mandible to the skull and performs the most complicated movement in the mouth and jaw system^1,2^. The overall prevalence of temporomandibular joint disorders (TMDs) is ~31% on adults and 11% for children^3^. Temporomandibular joint osteoarthritis (TMJOA) is now recognized as a highly prevalent subtype that affects from 18 to 85% of patients with TMDs^4^. As one of the most severe subtypes of TMDs, TMJOA is characterized by synovial inflammation, cartilage degradation and subchondral bone resorption^5^. It is a multifactorial joint disorder confounding with multiple factors including age, joint overload, trauma and psychological factors^6^. The clinical manifestations of the disease are varied, including joint clicking, joint pain, and restricted mouth opening, which seriously affects patients physically or mentally and their daily activities^7^. At present, the process of TMJOA therapy is hindered mainly by symptomatic treatment or surgical treatment, but neither of them can completely repair the damaged TMJ and restore its function. Therefore, it is of great significance to explore the unknown molecular mechanisms underlying TMJOA, and discover potential biomarkers and new therapeutic targets.

Inflammation is believed to be one of the most crucial factors in the pathological changes in TMJOA and is reported as a “triggering factor” of TMJOA^8,9^. An increasing number of studies have demonstrated that immune cell infiltration plays a critical role in TMJOA development. For example, it has been shown that increased number and infiltration of macrophages in the synovial lining are the main pathological features of synovitis, histologically characterized by thickening of synovial tissue. During TMJOA progression, synovial macrophages can be activated^10^. Additionally, it has been elucidated that inflammatory cytokines, including interleukin (IL)-1β, IL-6, IL-8, IL-17, and tumor necrosis factor (TNF)-α, are elevated in synovial fluid of TMJOA patients, which leads to the destruction of cartilage matrix^8,11,12^. Therefore, to alleviate or even cure TMJOA, it is of great significance to understand the molecular mechanism of the effect of inflammation on TMJOA.

TMJOA is currently diagnosed using a combination of clinical examinations, patient history, and imaging techniques^13^. Imaging techniques, such as X-rays, MRI, or CT scans, are commonly used to observe joint structure and identify degenerative changes^14^. However, these methods have limitations, including low sensitivity in detecting early-stage of TMJOA. Additionally, imaging techniques may not always reveal the extent of joint damage or inflammation. Therefore, there is an urgent need to find a method for the early diagnosis of TMJOA.

In the present study, we carried out a bioinformatics analysis of synovium samples from TMJOA patients and their controls with the curated dataset GSE205389. We identified differentially expressed genes and transcripts in mRNA and lncRNA level, and explore the function of them with enrichment analysis, protein–protein interaction and lncRNA-mRNA interaction networks, and immune infiltration detection. We found that immune-related and muscle system-related pathways were significantly enriched in TMJOA samples, identified potential inflammatory cytokines and chemokines markers for the early diagnosis of TMJOA, and observed a higher immune infiltration in TMJOA.

## Materials and methods

### Study design

This study used the datasets GSE205389 curated from the Gene Expression Omnibus (GEO). The differentially expressed genes and long non-coding RNAs were identified with R software^15^. Additionally, the Kyoto Encyclopedia of Genes and Genomes (KEGG) and Gene Ontology (GO) analyses were performed to elucidate the biological processes involved. Furthermore, a protein–protein interaction (PPI) network was constructed to identify hub genes, and the co-expression network between mRNA and lncRNA was constructed to check the potential regulation and function of lncRNA on protein-coding genes. Finally, the immune cell infiltration analysis was conducted.

### Data curation and preprocessing

The datasets GSE205389 was downloaded from the GEO (https://www.ncbi.nlm.nih.gov/geo/). The GSE205389 dataset was generated for Homo sapiens with expression profiling by array, which is based on the GPL20301 platform Illumina HiSeq. 4000. There were 10 samples included, five synovium samples from TMJOA patients (referred as the case group) and five synovium samples from patients with reducible anterior disc displacement (referred as the control group). The expression data was preprocessed and ready to be used in Fragments Per Kilobase of exon model per Million mapped fragments (FPKM) and read count level. In total, there were 20 308 genes of mRNA and 36 519 transcripts of lncRNA. We filtered the duplicated genes and transcripts, as well as the none expressed genes and transcripts, which were referred as the genes and transcript in the case group and the control group with the average read counts of all samples =0. In the end, there were 16,188 genes of mRNA and 3859 transcripts of lncRNA. These genes and transcripts were used in the further downstream analysis. Principal component analysis (PCA) of the expressed genes and transcripts was performed to check the expression quality of the processed data and the correlation among samples from the case and control group.

### Detection of differentially expressed genes of mRNA (DEGs) and transcripts of lncRNA (DETs)

The “DESeq. 2” package^16^ in R software was applied to detect DEGs and DETs with reads counts respectively for the mRNA genes and the lncRNA transcripts with samples from the case group and the control group. The significant differentiated genes and transcripts were defined with $\left|\log_{2}\mathrm{FoldChange}\right|>10$ and adjusted *P* value less than 0.05, which was selected with permutation tests for the robustness detection. The DEGs and DETs were annotated with the human gene annotation R package “org.Hs.eg.db”^17^. The R packages “ggplot2”^18^, “pheatmap”^19^, “ggsci”^20^, and “ggrepel”^21^ were used to illustrate the properties of DEGs and DETs.

### The enrichment analysis and functional annotation of DEGs

The Kyoto Encyclopedia of Genes and Genomes (KEGG) and Gene Ontology (GO) pathway databases^22^ were used for the enrichment analysis of DEGs with the R package “fgsea”^23^. The enrichment results were shown with the R packages “ggplot2”^18^ and “clusterProfiler”^24,25^.

### The protein–protein interaction (PPI) networks generation and the hub genes selection

The construction of the protein–protein interaction (PPI) network was performed using the STRING database (Search Tool for the Retrieval of Interacting Genes; https://string-db.org/)^26^. The Cytoscape data analyzer (https://cytoscape.org/) was used to identify functionally important proteins in the PPI network. For the PPI network, the species was set to ‘*Homo sapiens*’ with a minimum interaction score of greater than orequal to 0.7, which could significantly show the protein interaction with relatively lower false positive rate. This threshold was relatively strict and usually applied in the previous published work^27^. Nodes not connected within the network are not displayed. This process generates a network diagram and outputs the results of the protein–protein interactions. The top 10 hub genes were screened by using the Cytoscape app cytoHubba based on MCC algorithms. The Cytoscape plugin MCODE identified the most important modules of the network map.

### The lncRNA-mRNA co-expression network construction

The correlation between lncRNA and mRNA was pair wise detected by the Pearson correlation between DETs and DEGs. The R package “pheatmap”^19^ was used to check the correlation hub. The Cytoscape was used to draw the hub of the co-expression network. To further explore the potential function of the DEGs involved in the network hub, we conducted an enrichment analysis with Enrichr (REACTOME) (https://maayanlab.cloud/Enrichr/)^28^.

### Immune cell infiltrations identification

The immune cell infiltration analysis was conducted with CIBERSORTx and confirmed with xCells both in the case and the control groups^29^. The reference “LM22” was applied for the immune cell infiltrations identification. The *t*-test was applied to check the difference of infiltrations between the case and control groups.

## Results

### Principle components analysis of expressed genes in TMJOA samples with controls

**Figure 1:**
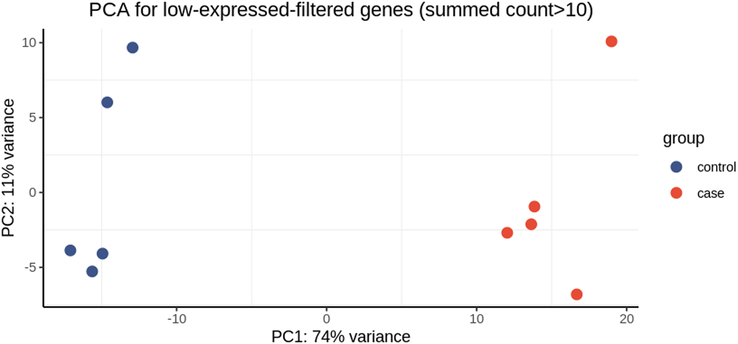
PCA in mRNA gene expression level. The *x* and *y*-axis indicated the principal component 1 and 2, respectively. The value of the coordinate axis represented the percentage of variance corresponding to the principal component interpretation population. The red indicated the samples with temporomandibular joint osteoarthritis and the blue indicated the controls. PCA, principal component analysis.

PCA was conducted with TMJOA samples and their controls to check the sample correlations in mRNA gene expression level, in which the expressed genes in samples were selected. The samples were separated into two groups based on the disease status of TMJOA. The coordinate axis indicated the principal component after dimensionality reduction, representing the main difference between samples. Respectively, PCA1 and PCA2 explained 74% and 11% of the total variation (Fig. 1).

### Differential expressed mRNA genes (DEGs) and lncRNA transcripts (DETs) identification

**Figure 2:**
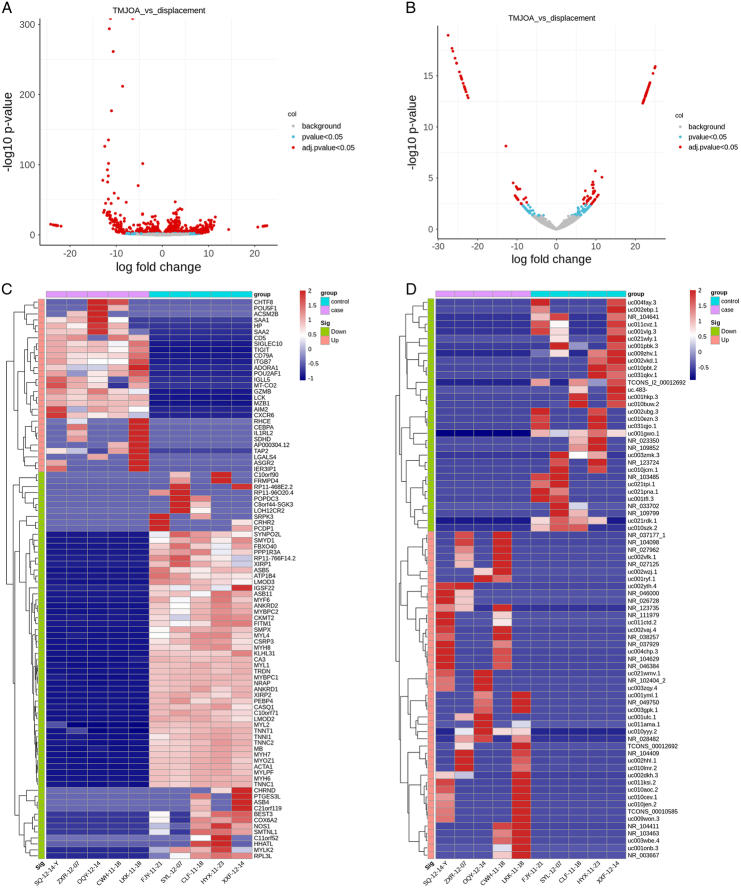
The illustration of differentially expressed genes of mRNA and transcripts of lncRNA. (A, B) In the volcano plots, the *x*-axis indicated the log 2 transformed fold changes and the y-axis indicated the −log10 transformed *P* values. Each dot indicated a gene or a transcript. The color indicated the significance level of the genes or transcripts. The gray dots indicated as “background” with *P* value ≥ 0.05 of the genes or transcripts; the green dots indicated the genes or transcripts with *P* value <0.05 but adjusted *P* value ≥ 0.05; and the red dots indicated the genes or transcripts with adjusted *P* value <0.05. (C, D) In the heatmaps, the genes or transcripts were clustered in the significance level. The case and control indicated the samples with TMJOA and the controls, respectively. TMJOA, temporomandibular joint osteoarthritis.

After data processing, we identified 171 DEGs and DETs. Respectively, there were 94 DEGs (29 up- and 65 down-regulated genes) and 77 DETs (45 up- and 32 down-regulated transcripts). The differential profiles were presented in volcano plots (Fig. 2A and B) and heatmaps (Fig. 2C and D) of DEGs and DETs, respectively.

### Functional exploration of DEGs

**Figure 3:**
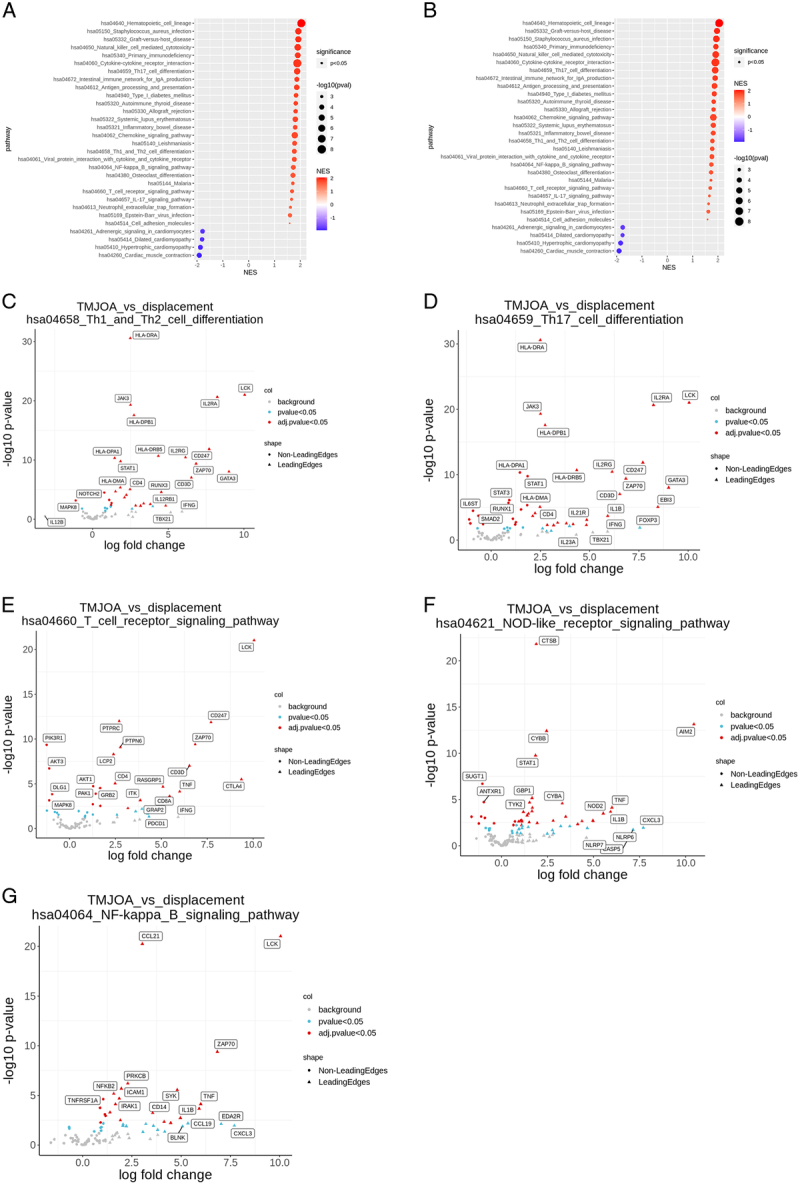
The illustration of top 30 results in differentially expressed genes of mRNA enrichment analysis and the expression profiles of the genes involved in the noted pathways. (A) The enrichment analysis results with Kyoto Encyclopedia of Genes and Genomes database. (B) The enrichment analysis results with Gene Ontology database. The size of the circle represented the −log10 transformed *P* value of the enrichments and the color of the circles indicated the enrichment degrees. (C, D) The expression profiles of the genes involved in Th1, Th2 (C) and Th17 (D) cell differentiation. (E–G) The expression profiles of the genes involved in T-cell receptor signaling pathway, NOD-like receptor signaling pathway and NF-kB signaling pathway. TMJOA, temporomandibular joint osteoarthritis.

We explored the function of DEGs with Kyoto Encyclopedia of Genes and Genomes (KEGG) and Gene Ontology (GO) with pathway enrichment analysis. For the KEGG enrichment analysis, the significant pathways were identified, including the cytokine-cytokine-receptor interaction, chemokine signaling pathway, natural killer cell-mediated cytotoxicity, Th17, Th1 and Th2 cell differentiation, T-cell receptor signaling pathway and NF-κB signaling pathway (Fig. 3A). For the GO enrichment analysis, the most significant enriched pathways included immune response, T-cell activation and differentiation, leukocyte cell activation, the muscle system process and the contractile fiber (Fig. 3B). In two enrichment profiles, immune-related pathways, especially for the T-cell activation and differentiation, were shown upregulated in samples with TMJOA, indicating the potential of the immune activation, especially for T cells functional activation in TMJOA disease progression. In the pathways of Th17, Th1 and Th2 cell differentiation and T-cell receptor signaling, we found that *LCK, ZAP70, IL2RA, IL2RG, CD247* and *CD3D* were significantly upregulated in TMJOA samples (Fig. 3C-E).

Additionally, according to the pathway enrichment analysis results, DEGs were significantly enriched in NOD-like receptor signaling pathway and NF-kB signaling pathway. For NOD-like receptor signaling pathway, the genes *AIM2, NOD2, IL1B,* and *TNF* were found to be higher expressed in TMJOA samples when compared with controls. For NF-κB signaling pathway, *LCK, ZAP70, IL1B,* and *TNF* were found to be higher expressed in TMJOA samples (Fig. 3F-G).

Beside of that, muscle system process-related pathways were significantly down-regulated in the two enrichment profiles, indicating the potential of muscle system dysfunction in TMJOA samples, which could probably contribute to the disease progression.

### Protein–protein interaction network construction

**Figure 4:**
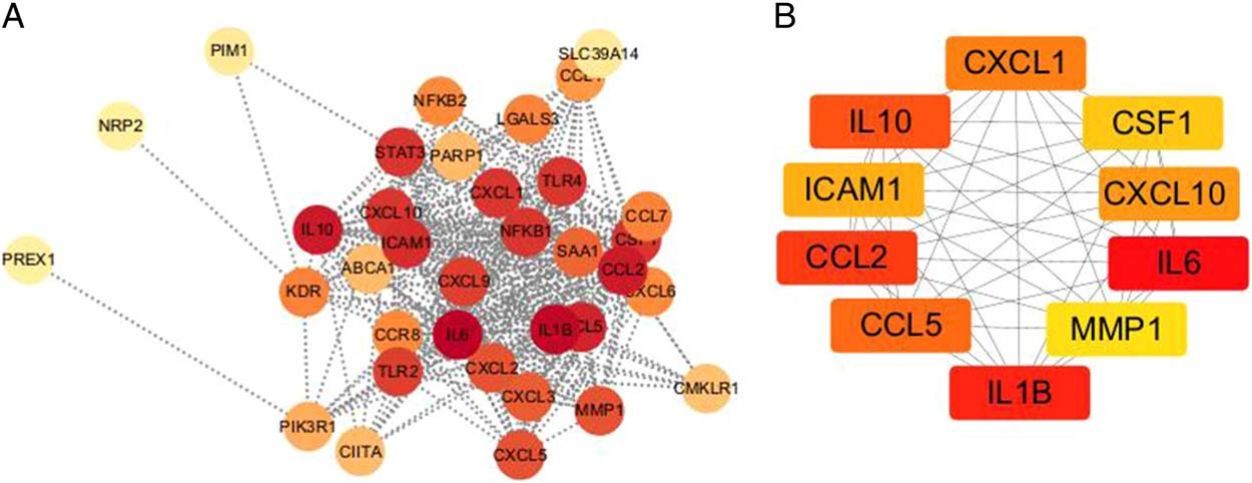
Protein–protein interaction network. (A) The PPI network was constructed using the STRING database. The key interaction network of the mRNA genes was showed, which reflected that these genes were highly correlated and might have a significant interaction with potential regulatory functions. Each node indicated one gene and the dashed lines between genes indicated the interaction. (B) The top 10 hub genes were identified with the cytoHubba plugin, which was the key genes in the PPI network. Each node indicated one gene and the lines between genes indicated the interaction. PPI, protein–protein interaction.

The PPI network was constructed with the DEGs using the STRING database and Cytoscape. In total, there were 35 nodes and 320 edges contained in the module and the average nodal degree was 9.14 (Fig. 4A). These genes were highly correlated and might have the potential regulatory functions, which could be highly activated and should be further explored in the function analysis. And among them, the top 10 hub genes were identified, including *IL6, IL1B, CCL2, IL10, CCL5, CXCL1, CXCL10, ICAM1, CSF1* and *MMP1*, with the cytoHubba plugin (Fig. 4B). The genes were mainly involved in the regulation of inflammatory response, indicating the inflammatory-related process existing in TMJOA samples as mentioned in above enrichment analysis and the potential immune infiltration existing in TMJOA samples, reflecting the consistency of our analysis.

### The lncRNA and mRNA co-expression network construction

**Figure 5:**
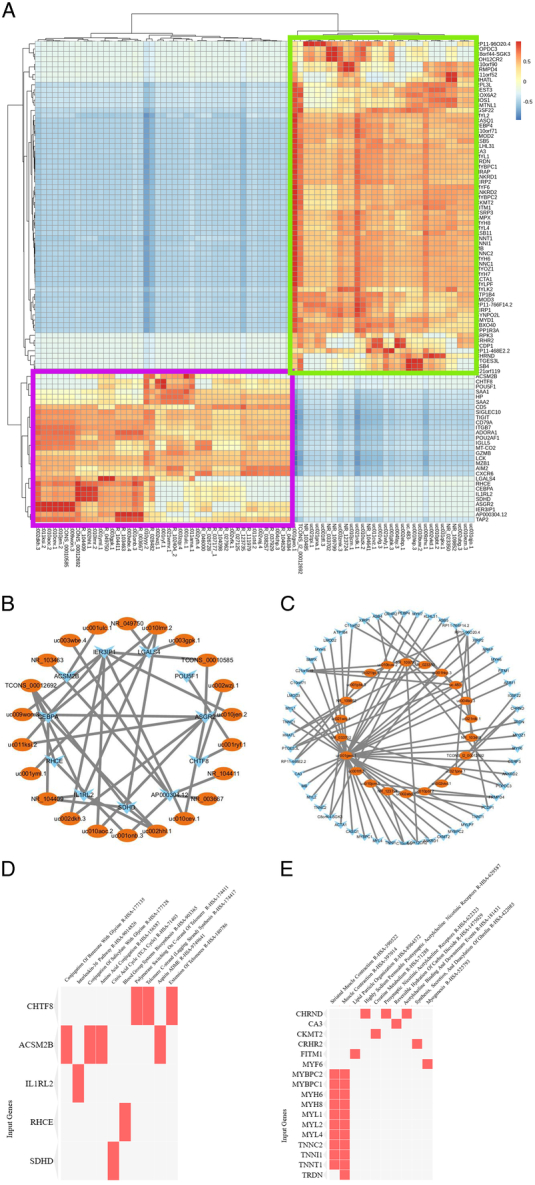
LncRNA-mRNA co-expression network properties. (A) LncRNA-mRNA co-expression network. The *x*-axis indicated the lncRNA transcripts of lncRNA (DETs) and the *y*-axis indicated the mRNA DEGs. The pair wise correlation was conducted with Pearson correlation. The color indicated the correlation degree between each differentially expressed genes of mRNA (DEG) and DET pair. In the color bar, the red indicated the positive correlation, and the blue indicated the negative correlation. The positive correlation patterns were highlighted in purple and green. (B, C) The network diagram of DEGs and DETs in the red and yellow highlighted patterns respectively. The blue V-shape nodes represented mRNA DEGs and the orange oval nodes represented lncRNA DETs. The lines indicated the co-expression correlation between DEGs and DETs. (D, E) The enrichment analysis of DEGs in the purple and green highlighted patterns with REACTOME.

The protein-coding DEGs and lncRNA DETs were used to conduct the co-expression network to check the potential interaction and regulation between lncRNA and mRNA. As shown in Figure 5A, two major clusters were identified. In the cluster highlighted in purple, there were 22 lncRNA positively correlated with 11 DEGs. While in the cluster highlighted in green, there were 22 lncRNA positively correlated with 36 DEGs. We used these DEGs in the two highlighted clusters to construct the lncRNA-mRNA co-expression network. The network diagram contained 33 nodes, 40 edges and 76 nodes, 73 edges, respectively, for the left and right clusters (Fig. 5B and C). The co-expressed protein coding RNA and lncRNA pairs indicated the potential regulatory effects for TMJOA progression.

To further explore the potential regulatory function of the co-expression pairs, we selected the top correlated lncRNA-mRNA pair to conducted the functional enrichment analysis with REACTOME database. We found that in the purple cluster, the lncRNA-correlated DEGs were significantly enriched in conjugation of benzoate with glycine, interleukin-36 pathway and conjugation of salicylate with glycine pathways (Fig. 5D), indicating the function of inflammatory response in the progression of TMJOA. And for the green cluster, the lncRNA-correlated DEGs were significantly enriched in striated muscle contraction, muscle contraction and lipid particle organization pathways (Fig. 5E), indicating the function of muscle-related pathways in TMJOA.

### Immune cell infiltrations detection in TMJOA samples and controls

**Figure 6:**
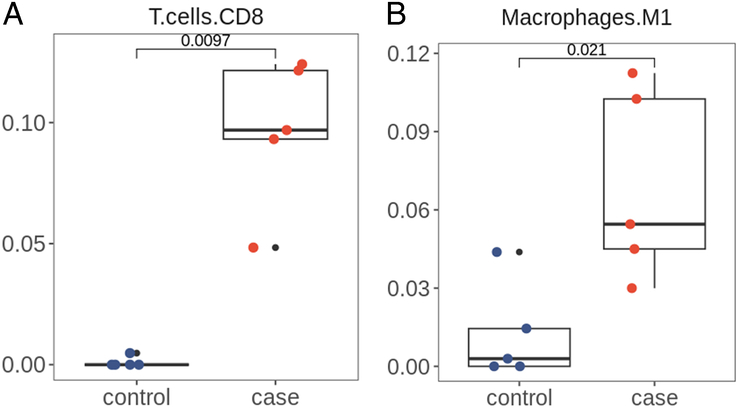
Immune cell infiltrations analysis of temporomandibular joint osteoarthritis (TMJOA). (A) The imputed infiltrates of CD8^+^ T cells in TMJOA samples (case) and controls (control). (B) The imputed infiltrates of macrophages in TMJOA samples (case) and controls (control). Each dot indicated a sample. The comparison test was conducted with *t*-test.

**Figure 7:**
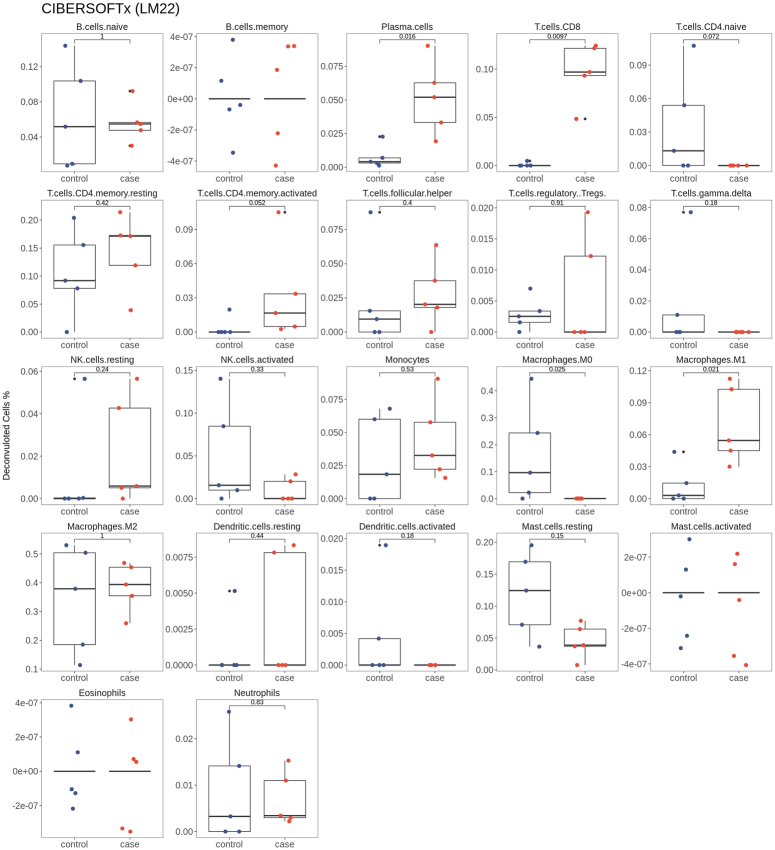
The immune cell infiltration results detected with CIBERSORTx.

**Figure 8:**
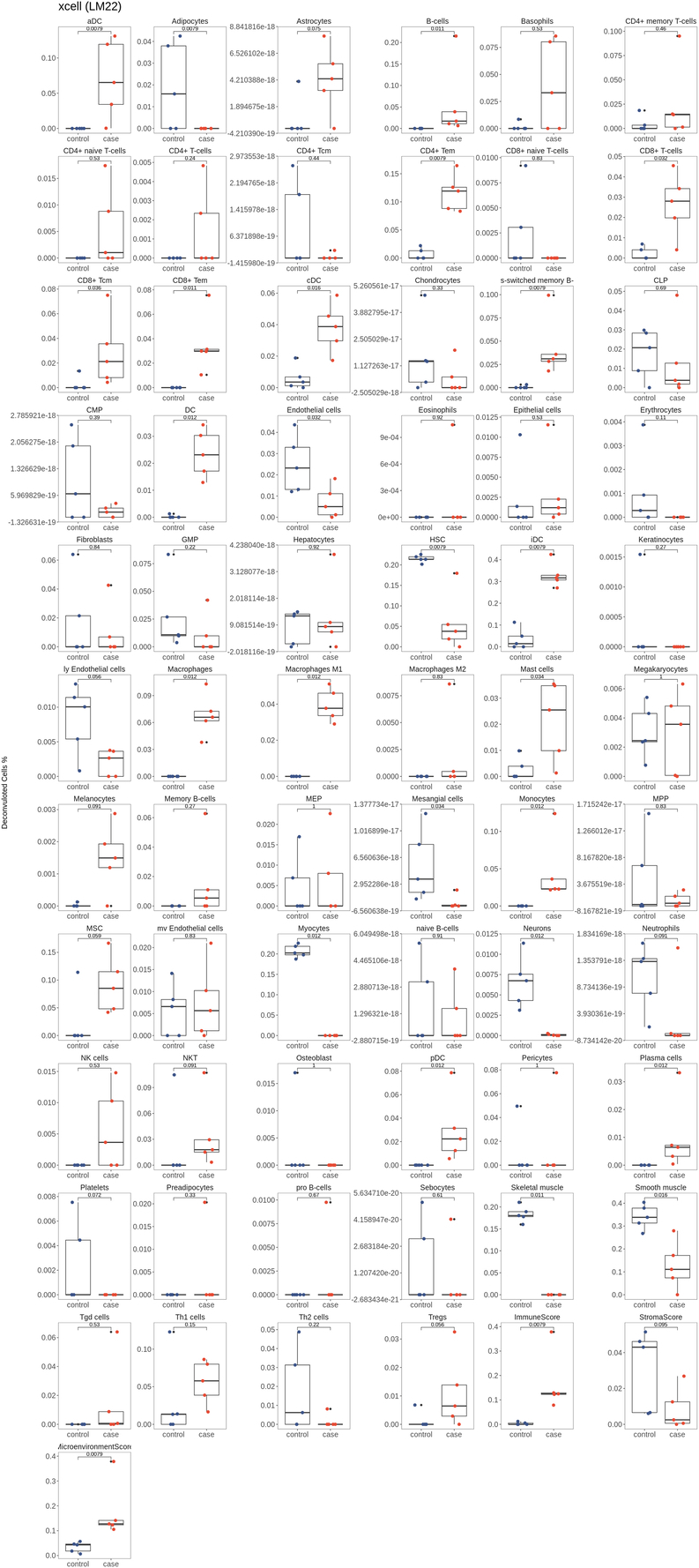
The immune cell infiltration results confirmed with xCells.

The immune cell infiltration was detected with CIBERSORTx and confirmed with xCells. The difference of the infiltrations was compared between TMJOA samples and healthy controls. We found that CD8^+^ T cells and M1 macrophages infiltrations were significantly increased in TMJOA samples when compared with that in controls (Figs. 6–8).

## Discussion

TMJOA is one of the most severe types of TMD. Although patients with end-stage TMJOA are advised to undergo total joint replacement, the high cost of TMJ prostheses severely restricts their use in most developing countries, which has caused a huge social and economic burden^30,31^.

Synovial inflammation has been proven to play a key role in the incidence and development of TMJOA^32^. Vascular proliferation, fibrosis, and inflammatory cell infiltration of surrounding synovial tissue can destroy the TMJ condylar cartilage^6^. Previous studies mainly focus on the pathologic changes of condylar cartilage or subchondral bone on the progression of TMJOA. However, the changes in surrounding synovial tissues and their effects on TMJOA have been largely neglected. To date, there have been relatively few studies that use public data of TMJOA for bioinformatics analysis. Most of previous published studies focused on the expression profiles description of TMJOA in the human cell line level or in mouse model, or just in specific pathways.

In the present study, we carried out a bioinformatics analysis of synovium samples from TMJOA patients and their controls. Differentially expressed mRNA genes and lncRNA transcripts (DEGs and DETs) were identified between TMJOA samples and their controls. And the Enrichment analysis and protein–protein interaction analysis were applied to explore the potential function of the identified DEGs, showing that the DEGs were closely related to immune response and the muscle system process.

Then, the co-expression network was constructed between mRNA and lncRNA to check the potential regulation and function of lncRNA on protein-coding genes. Finally, immune infiltrations were checked to confirm our analysis above.

In general, our work is solid and robust. For the enrichment analysis, we found that the previously reported immune-related signaling pathways in TMJOA also showed a significant enrichment in our analysis, which have the potential to mediate the immune activation in TMJOA progression. Our results were consistent with the study of Lou *et al.* ^33^, which conducted a bioinformatics analysis of synovial fluid-derived mesenchymal stem cells with and without IL-1β stimulation, as well as Monasterio *et al.* ^34^, which demonstrated that cytokines associated with the Th1/Th17/Th22 axis of immuno-inflammatory response were involved in TMJOA pathogenesis. Except for the pathways mentioned above, the NOD-like receptors signaling pathway and NF-κB signaling pathway were also significantly upregulated in TMJOA according to our analysis. The NOD-like receptors signaling pathway has been confirmed recently to participate in the synovitis of TMJOA^35^. Targeting NOD-like receptor protein 3 (*NLRP3*) alleviated synovial inflammation-induced chondrocyte pyroptosis and cartilage degradation during TMJOA^36^. Also NF-κB is a crucial transcription factor in inflammatory and immune responses^37–39^ and NF-κB signaling pathway is more commonly reported in the progression of TMJOA^40^. All the results above indicated the potential role of immune response in TMJOA.

For the PPI network construction, we screened the top 10 hub genes, including *IL6, IL1B, IL10, CCL2, CCL5, CXCL1, CXCL10, ICAM1, CSF1* and *MMP1* (Fig. 4B), which are mainly involved in pathways related to inflammation, immune responses, and tissue remodeling, including the MAPK pathway, NF-κB pathway, JAK/STAT pathway, and PI3K/AKT pathway^41^. During the pathogenesis of TMJOA, chemokines such as *CCL2, CCL5, CXCL1, CXCL10* can promote the infiltration of inflammatory cells in TMJ, causing the release of degradation enzymes and inflammatory cytokines. At the same time, inflammatory cytokines like IL-6 and IL-1β, also stimulate synovial fibroblasts to produce more chemokines^42^. The genes *ICAM1* and *CSF1* are also common pro-inflammatory cytokines that have a remarkable role in the development and progression of TMJOA^43,44^. As for *MMP-1*, which is released by synovial fibroblasts and chondrocytes, promotes cartilage degradation^45^. Therefore, the biomarkers we found might help to make an early diagnosis for TMJOA, especially for patients with mild symptoms. Also, small molecule inhibitors or antibodies that selectively target the inflammatory cytokines or pathways can be used to impede the progression of early-stage TMJOA in clinic.

For the immune infiltrations, we showed that CD8^+^ T cells and M1 macrophage infiltration were increased in TMJOA sample, which was consistent with our enrichment results. In addition, as the main source of inflammatory enzymes and cytokines, synovial macrophages play a vital role in the development of TMJOA^46,47^. There is a dramatic increase in the number of M1 macrophages in the inflamed synovium^48^ and inhibition of M1 macrophage polarization alleviates TMJOA^49^. The increased infiltration of CD8^+^ T cells and M1 macrophages in TMJOA suggests an enhanced immune response and an inflammatory environment. To selectively reduce the activity of CD8^+^ T cells or M1 macrophages without influencing other immune cells, potential strategies could be considered for future TMJOA treatment, including monoclonal antibodies of CD8^+^ T cells or M1 macrophages, small molecule inhibitors targeting specific activation pathways, or gene editing technologies to modify key genes.

Finally, for the lncRNA-mRNA co-expression, we identified two clusters for DEGs and DETs correlated pairs, which was mainly enriched in inflammatory and muscle-related pathways, indicating a significant interaction between lncRNAs and mRNAs involved in those pathways. These interactions suggest that lncRNAs may play a regulatory role in the expression of genes critical to TMJOA pathogenesis, providing a potential new area for TMJOA treatment. In recent years, lncRNA has been reported to be involved in inflammatory responses of TMJOA, leading to the release of pro-inflammatory cytokines and matrix-degrading enzymes. For instance, Wu *et al.* ^50^ demonstrated that LncRNA-EPS regulated the inflammatory process of condylar chondrocytes and alleviated TMJOA by binding to SRSF3. Additionally, the knockdown of lncRNA AK094629 attenuates IL‑1β induced IL‑6 expression in synovium‑derived mesenchymal stem cells from the TMJ^51^. All the evidence above suggests that lncRNA might serve as a potential target for TMJOA treatment. We also identified the differential expressed lncRNA for TMJOA, which would be further confirmed in the experimental analysis. Therefore, the significance for the lncRNA-mRNA interactions identification could help us to identify the targets involved in the inflammatory response and muscle-related pathways, which could be the biomarkers for the TMJOA therapy, diagnosis and prognosis prediction, giving an insight into the further biomarkers’ validation in preclinic and clinic.

One limitation of this study is the lack of functional experiments to validate the bioinformatics analysis results, which is on the way. Additionally, the sample size could be expanded to get more accurate results and ensure a broader applicability of the findings.

## Conclusions

In conclusion, we detected the poetical differential expressed mRNA genes and lncRNA transcripts for TMJOA, and identified the potential inflammatory cytokines and chemokines markers for TMJOA diagnosis, as well as the immune infiltrations of CD8^+^ T cells and M1 macrophages in TMJOA, indicating the occurrence and progression mechanisms of TMJOA. By providing potential inflammatory markers, our findings could also contribute to the early diagnosis of TMJOA and can help to customize personalized treatment strategies for TMJOA patients in the future. However, translating these findings into clinical practice might be limited by the need for further experimental validation.

## Ethics approval

Not applicable.

## Consent

Not applicable.

## Source of funding

This work was supported by grants from the Project of Shanghai Municipal Health Commission (grant no. 20214Y0080), Xuhui Municipal Health Commission (grant no. SHXH202210) and the Medical Key Subject of Xuhui District (grant no. SHXHZDXK202302).

## Author contribution

M.Z., M.X. and R.S. contributed to conception, design, data acquisition, analysis, and interpretation, drafted and critically revised the manuscript; M.L. helped perform the bioinformatics analysis and critically revised the manuscript. W.Q. and M.F. contributed to conception, design, data interpretation and critically revised the manuscript. All authors gave final approval and agreed to be responsible for all aspects of the work.

## Conflicts of interest disclosure

The authors declare that they have no competing interests.

## Research registration unique identifying number (UIN)

Research registration was not required for this study.

## Guarantor

Mingyue Fan.

## Data availability statement

The datasets used and/or analyzed during the current study are available from the corresponding author upon reasonable request.

## Provenance and peer review

Not commissioned, externally peer-reviewed.
